# Synthesis of trifluoromethyl-substituted pyrazolo[4,3-*c*]pyridines – sequential versus multicomponent reaction approach

**DOI:** 10.3762/bjoc.10.183

**Published:** 2014-07-31

**Authors:** Barbara Palka, Angela Di Capua, Maurizio Anzini, Gyté Vilkauskaité, Algirdas Šačkus, Wolfgang Holzer

**Affiliations:** 1 Division of Drug Synthesis, Department of Pharmaceutical Chemistry, Faculty of Life Sciences, University of Vienna, Althanstrasse 14, A-1090 Vienna, Austria; 2 Department of Biotechnology, Chemistry and Pharmacy, University of Siena, I-53100, Siena, Italy; 3 Institute of Synthetic Chemistry, Kaunas University of Technology, LT-50254 Kaunas, Lithuania

**Keywords:** microwave-assisted reactions, multicomponent reactions, NMR (^1^H, ^13^C, ^15^N, ^19^F), Sonogashira coupling, trifluoromethylpyrazoles

## Abstract

A straightforward synthesis of 6-substituted 1-phenyl-3-trifluoromethyl-1*H*-pyrazolo[4,3-*c*]pyridines and the corresponding 5-oxides is presented. Hence, microwave-assisted treatment of 5-chloro-1-phenyl-3-trifluoromethylpyrazole-4-carbaldehyde with various terminal alkynes in the presence of *tert*-butylamine under Sonogashira-type cross-coupling conditions affords the former title compounds in a one-pot multicomponent procedure. Oximes derived from (intermediate) 5-alkynyl-1-phenyl-3-trifluoromethyl-1*H*-pyrazole-4-carbaldehydes were transformed into the corresponding 1*H*-pyrazolo[4,3-*c*]pyridine 5-oxides by silver triflate-catalyzed cyclization. Detailed NMR spectroscopic investigations (^1^H, ^13^C, ^15^N and ^19^F) were undertaken with all obtained products.

## Introduction

**Figure 1:**
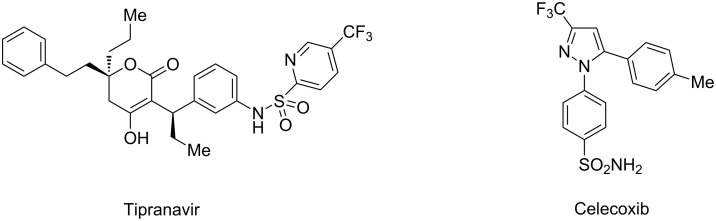
Important drug molecules containing a trifluoromethylpyridine, respectively a trifluoromethylpyrazole moiety.

Fluorine-containing compounds play an important role in medicinal and pharmaceutical chemistry as well as in agrochemistry [1–4]. A popular approach for the modulation of activity consists in the introduction of one or more fluorine atoms into the structure of a bioactive compound. This variation frequently leads to a higher metabolic stability and can modulate some physicochemical properties such as basicity or lipophilicity [1–2]. Moreover, incorporation of fluorine often results in an increase of the binding affinity of drug molecules to the target protein [1–2]. As a consequence, a considerable amount – approximately 20% – of all the pharmaceuticals being currently on the market contain at least one fluorine substituent, including important drug molecules in different pharmaceutical classes [5]. Keeping in mind the above facts, the synthesis of fluorinated heterocyclic compounds, which can act as building blocks for the construction of biologically active fluorine-containing molecules, is of eminent interest. In the field of pyrazoles, pyridines and condensed systems thereof trifluoromethyl-substituted congeners can be found as partial structures in several pharmacologically active compounds. In the pyridine series the HIV protease inhibitor Tipranavir (Aptivus^®^) [6] may serve as an example, within the pyrazole-derived compounds the COX-2 inhibitor Celecoxib (Celebrex^®^) is an important representative (Figure 1) [7].

In continuation of our program regarding the synthesis of fluoro- and trifluoromethyl-substituted pyrazoles and annulated pyrazoles [8–9] we here present the synthesis of trifluoromethyl-substituted pyrazolo[4,3-*c*]pyridines. The latter heterocyclic system represents the core of several biologically active compounds, acting, for instance, as SSAO inhibitors [10], or inhibitors of different kinases (LRRK2 [11–12], TYK2 [13], JAK [14–15]).

## Results and Discussion

### Chemistry

The construction of the pyrazolo[4,3-*c*]pyridine system can be mainly achieved through two different approaches. One strategy involves the annelation of a pyrazole ring onto an existing, suitable pyridine derivative [16]. Alternatively, the bicyclic system can be accessed by pyridine-ring formation with an accordant pyrazole precursor. Employing the latter approach we recently presented a novel method for the synthesis of the pyrazolo[4,3-*c*]pyridine system by Sonogashira-type cross-coupling reaction of easily obtainable 5-chloro-1-phenyl-1*H*-pyrazole-4-carbaldehydes with various alkynes and subsequent ring-closure reaction of the thus obtained 5-alkynyl-1*H*-pyrazole-4-carbaldehydes in the presence of *tert*-butylamine [17]. Furthermore, we showed that the oximes derived from the before mentioned 5-alkynylpyrazole-4-carbaldehydes can be transformed into the corresponding 1-phenylpyrazolo[4,3-*c*]pyridine 5-oxides [17].

**Scheme 1:**
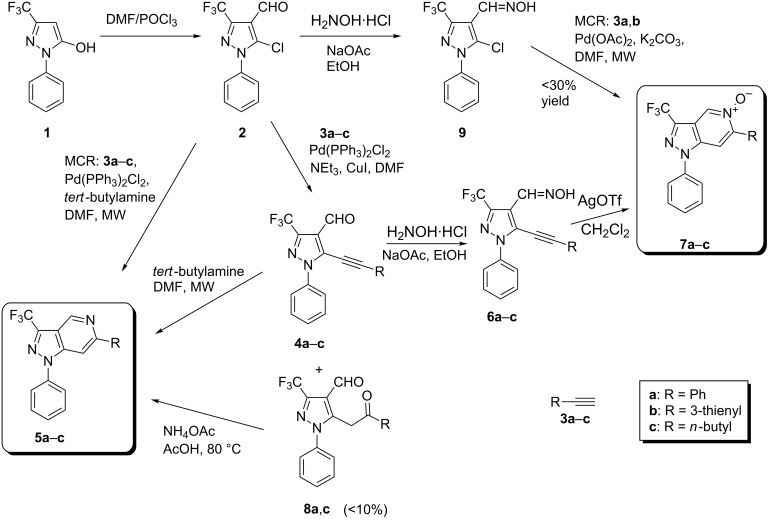
Synthesis of the title compounds.

For the synthesis of the title compounds a similar approach was envisaged. As starting material the commercially available 1-phenyl-3-trifluoromethyl-1*H*-pyrazol-5-ol (**1**) was employed which, after Vilsmaier formylation [18] and concomitant transformation of the hydroxy function into a chloro substituent by treatment with excessive POCl_3_, gave the chloroaldehyde **2** [19] (Scheme 1). Although Sonogashira-type cross-coupling reactions are preferably accomplished with iodo(hetero)arenes – considering the general reactivity I > Br/OTf >> Cl [20] – from related examples it was known that the chloro atom in 5-chloropyrazole-4-aldehydes is sufficiently activated to act as the leaving group in such kind of C–C linkages [17]. Indeed, reaction of chloroaldehyde **2** with different alkynes **3a**–**c** under typical Sonogashira reaction conditions afforded the corresponding cross-coupling products **4a**–**c** in good yields (Scheme 1). In some runs compounds of type **8** were determined as byproducts in differing yields, but mostly below 10%, obviously resulting from addition of water to the triple bond of **4** under the reaction conditions (or during work-up) and subsequent tautomerization of the thus formed enoles into the corresponding ketones. The hydration of C–C triple bonds under the influence of various catalytic systems, including also Pd-based catalysts, is a well-known reaction [21–22]. It should be emphasized that NMR investigations with compounds **8a**,**c** unambiguously revealed the methylene group adjacent to the pyrazole nucleus and the carbonyl moiety attached to the substituent R originating from the employed alkyne.

In the next reaction step, alkynylaldehydes **4a**,**b** were cyclized into the target pyrazolo[4,3-*c*]pyridines **5a**,**b** in 71%, resp. 52% yield by reaction with *tert*-butylamine under microwave assistance [17]. In view of the fact, that the two-step conversion **2**→**4a**,**b**→**5a**,**b** was characterized by only moderate overall yields (59%, resp. 43%) it was considered to merge these two steps into a one-pot multicomponent reaction. The latter type of reaction attracts increasing attention in organic chemistry due to its preeminent synthetic efficiency, also in the construction of heterocyclic and condensed heterocyclic systems [23–27]. After testing different reaction conditions we found that microwave heating of the chloroaldehyde **2** with *tert*-butylamine in the presence of 6 mol % of Pd(PPh_3_)_2_Cl_2_ afforded the desired pyrazolopyridines **5a**–**c** in high (**5a**: 89%, **5c**: 92%) respectively acceptable yields (**5b**: 51%) in a single one-pot and copper-free reaction step (Scheme 1). It should be mentioned that compounds **5** are also accessible by heating of ketones **8** with ammonium acetate in acetic acid according to a procedure described in [28]. Following this way, **5a** and **5c** were obtained in 70% yield from the corresponding ketones **8a** and **8c**. Although ketones **8** were only obtained as byproducts, the latter transformation allowed increasing the overall yield of compounds **5** through this ‘bypass’.

In order to gain access to the corresponding *N*-oxides of type **7**, aldehydes **4a**–**c** were transformed into the corresponding oximes **6a**–**c** by reaction with hydroxylamine hydrochloride in ethanol in the presence of sodium acetate (Scheme 1). Subsequent treatment of the oximes with AgOTf in dichloromethane [29] finally afforded the corresponding pyrazolo[4,3-*c*]pyridine 5-oxides **7a**–**c** by a regioselective 6-*endo-dig* cyclisation [30] in high yields. Moreover, we tested an alternative approach to access compounds **7** through multicomponent reactions (MCR). Attempts to react chloroaldehyde **2** with hydroxylamine hydrochloride and an alkyne **3** in the presence of a suitable catalytic system were not successful. However, after conversion of **2** into the corresponding aldoxime **9** the latter could be transformed into the *N*-oxides **7a** and **7b** by reaction with alkynes **3a** and **3b**, respectively, employing Pd(OAc)_2_ as the catalyst and under microwave irradiation (Scheme 1). Although a number of different reaction conditions were tested, we were not able to increase the yields in excess of 30%. Thus, with respect to the overall yields the successive approach **2**→**4**→**6**→**7** (overall yields: **6a**: 62%, **6b**: 43%) here is still advantageous compared to the multicomponent reaction following the path **2**→**9**→**7**. Azine *N*-oxides of type **7** are estimated to be of particular interest due to the possibility of further functionalization adjacent to the nitrogen atom (position 4), for instance by palladium-catalyzed direct arylation reactions [31].

### NMR spectroscopic investigations

In Supporting Information File 1 the NMR spectroscopic data of all compounds treated within this study are indicated. Full and unambiguous assignment of all ^1^H, ^13^C, ^15^N and ^19^F NMR resonances was achieved by combining standard NMR techniques [32], such as fully ^1^H-coupled ^13^C NMR spectra, APT, HMQC, gs-HSQC, gs-HMBC, COSY, TOCSY, NOESY and NOE-difference spectroscopy.

In compounds **4**–**7** the trifluoromethyl group exhibits very consistent chemical shifts, ranging from δ(F) −60.8 to −61.9 ppm. The fluorine resonance is split into a doublet by a small coupling (0.5–0.9 Hz) due to a through-space (or possibly ^5^*J*) interaction with spatially close protons (**4**: CHO; **6**: CH=N; **5** and **7**: H-4). Reversely, the signals of the latter protons are split into a quartet (not always well resolved). The corresponding carbon resonance of CF_3_ is located between 120.2 and 121.2 ppm with the relevant ^1^*J*(C,F) coupling constants being approximately 270 Hz (269.6–270.6 Hz). As well, the signal of C-3 is always split into a quartet (*J* ~ 40 Hz) due to the ^2^*J*(C,F_3_) coupling.

As the ^15^N NMR chemical shifts were determined by ^15^N,^1^H HMBC experiments the resonance of (pyrazole) N-2 was not captured owing to the fact that this nitrogen atom lacks of sufficient couplings to protons, thus disabling the necessary coherence transfer (^19^F,^15^N HMBC spectra were not possible with the equipment at hand). For N-1, with pyrazole derivatives **4** and **6** remarkably larger ^15^N chemical shifts were detected (−158.8 to −160.2 ppm) compared to the corresponding signals for pyrazolopyridines **5** and **7** (−182.2 to −185.9 ppm). When switching from an azine to an azine oxide partial structure (**5**→**7**) the N-5 resonance exhibits an explicit upfield shift (15.6–18.3 ppm), being typical for the changeover from pyridine to pyridine *N*-oxide [33].

NMR experiments also allowed the determination of the stereochemistry of oximes **6**: considering the size of ^1^*J*(N=C-H) which is strongly dependent on lone-pair effects [34] as well as the comparison of chemical shifts with those of related, unambiguously assigned oximes [17] reveals *E*-configuration at the C=N double bond.

With byproduct **8a** the position of the carbonyl group unequivocally follows from the correlations between phenyl protons and the carbonyl C-atom and, reversely, from those between the methylene protons with pyrazole C-4 and pyrazole C-5 (determined by ^13^C,^1^H HMBC).

**Figure 2:**
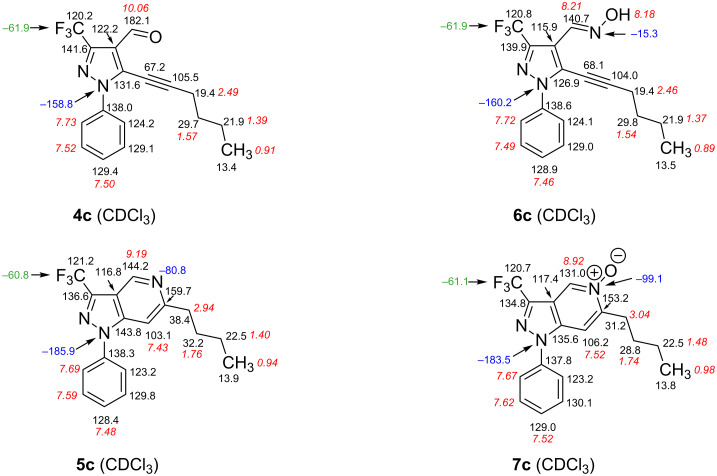
^1^H (in italics, red), ^13^C (black), ^15^N (in blue) and ^19^F NMR (green) chemical shifts of compounds **4c**, **5c**, **6c** and **7c** (in CDCl_3_).

In Figure 2 essential NMR data for the complete series of type **c** (**4c**, **5c**, **6c**, **7c**) are displayed, which easily enables to compare the notable chemical shifts and allows following the trends described above.

Full experimental details as well as spectral and microanalytical data of the obtained compounds are presented in Supporting Information File 1.

## Conclusion

To sum up, the presented approach represents a simple method for the synthesis of 6-substituted 1-phenyl-3-trifluoromethyl-1*H*-pyrazolo[4,3-*c*]pyridines **5** and the analogous 5-oxides **7** starting from commercially available 1-phenyl-3-trifluoromethyl-1*H*-pyrazol-5-ol (**1**). In the case of the former (**5**) the described multicomponent reaction approach is superior compared to the sequential one, whereas the step-by-step synthesis of *N*-oxides **7** is still characterized by higher overall yields. In addition, in-depth NMR studies with all synthesized compounds were performed, affording full and unambiguous assignment of ^1^H, ^13^C, ^15^N and ^19^F resonances and the designation of ascertained heteronuclear spin-coupling constants.

## Supporting Information

File 1 Experimental details and characterization data.
