# Supplementary material for: Synthesis of trifluoromethyl-substituted pyrazolo[4,3-c]pyridines – sequential versus multicomponent reaction approach
Source: Beilstein J Org Chem. 2014 Jul 31;10:1759–64. doi: 10.3762/bjoc.10.183 (PMC4142841; doi:10.3762/bjoc.10.183)
Supplement: File 1 — Experimental details and characterization data. [file Beilstein_J_Org_Chem-10-1759-s001.pdf]

Supporting Information

for

Synthesis of trifluoromethyl-substituted pyrazolo[4,3-*c*]pyridines – sequential versus multicomponent reaction approach

Barbara Palka[1], Angela Di Capua[1,2], Maurizio Anzini[2], Gyté Vilkauskaité[1,3], Algirdas Šačkus[3] and Wolfgang Holzer,[1]*

Address: [1]Division of Drug Synthesis, Department of Pharmaceutical Chemistry, Faculty of Life Sciences, University of Vienna, Althanstrasse 14, A-1090 Vienna, Austria, [2]Department of Biotechnology, Chemistry and Pharmacy, University of Siena, I-53100, Siena, Italy and [3]Institute of Synthetic Chemistry, Kaunas University of Technology, LT-50254 Kaunas, Lithuania

Email: Wolfgang Holzer* - wolfgang.holzer@univie.ac.at

*Corresponding author

Experimental details and characterization data

1. General

Melting points were determined on a Reichert–Kofler hot-stage microscope and are uncorrected. Mass spectra were obtained on a Shimadzu QP 1000 instrument (EI, 70 eV) and on a Bruker maXis 4G instrument (ESI-TOF, HRMS). IR spectra were recorded on a Perkin-Elmer FTIR 1605 spectrophotometer. ^{1}H, ^{13}C, ^{15}N and ^{19}F NMR spectra were recorded with a Bruker Avance III 400 spectrometer (400 MHz for ^{1}H, 100 MHz for ^{13}C, 40 MHz for ^{15}N, 376 MHz for ^{19}F) or a Bruker Avance 500 spectrometer (500 MHz for ^{1}H, 125 MHz for ^{13}C, 50 MHz for ^{15}N, 470 MHz for ^{19}F) at 297 K using "directly" detecting broadband observe (BBFO) probes. The center of the solvent signal was used as an internal standard which was related to TMS with δ 7.26 ppm (^{1}H in $CDCl_3$) and δ 77.0 ppm (^{13}C in $CDCl_3$). ^{15}N NMR spectra (gs-HMBC, gs-HSQC) were referenced against neat, external nitromethane, ^{19}F NMR spectra by absolute referencing via Ξ ratio. Digital resolutions were 0.25 Hz/data point in the

^1H and 0.4 Hz/data point in the ^1H-coupled ^{13}C-NMR spectra (gated decoupling). For the microwave reactions an Anton Paar Synthos 3000 oven was employed. For column chromatographic separations Merck Kieselgel 60 (70-230 mesh) was used. Light petroleum refers to the fraction with boiling point 40–65 °C. Yields are not optimized.

*2. 5-Chloro-1-phenyl-3-(trifluoromethyl)-1*H*-pyrazole-4-carbaldehyde (**2**)*

Phosphorus oxychloride (21.47 g, 140.0 mmol) was slowly added to dimethylformamide (2.56 g, 35.0 mmol) at 0 °C while stirring, then 1-phenyl-3-(trifluoromethyl)-1*H*-pyrazol-5-ol (**1**) (8.00 g, 35.0 mmol) was added and the mixture was heated at 95 °C for 4.5 h. After cooling to rt, it was slowly poured into ice-water (200 mL) under vigorous stirring, in the process an orange oil was formed. The mixture was extracted with EtOAc (3×50 mL), the combined organic phases were washed with brine, 5% aq $NaHCO_3$ solution and brine (each 30 mL), dried over anhydrous Na_2SO_4 and evaporated under reduced pressure. The residue was subjected to column chromatography (SiO_2, EtOAc/light petroleum 1:1) to afford 8.65 g (90 %) of a colorless oil which slowly solidified while standing: mp 34–36 °C. ^1H NMR (500 MHz, $CDCl_3$): δ 7.56 (m, 5H, Ph H), 10.06 (s, 1H, CHO) ppm. ^{13}C NMR (125 MHz, $CDCl_3$): δ 116.6 ($^2J_{C4,CHO}$ = 28.9 Hz, C-4), 120.1 (q, $^1J_{CF3}$ = 270.7 Hz, *C*F$_3$), 125.5 (Ph C-2,6), 129.5 (Ph C-3,5), 130.3 (Ph C-4), 133.7 ($^3J_{C5,CHO}$ = 3.3 Hz, C-5), 136.1 (Ph C-1), 142.9 ($^2J_{C3,CF3}$ = 40.0 Hz, $^3J_{C3,CHO}$ = 2.0 Hz, C-3), 181.1 (^1J = 183.9 Hz, *C*HO) ppm. ^{19}F NMR (470 MHz, $CDCl_3$): δ −61.9 (CF_3) ppm. IR (KBr): ν = 1700 (C=O) cm^{-1}. EIMS, *m/z* (rel. int.): 276 (28), 275 (44), 274 (80) $[M]^+$, 273 (100), 255 (4), 245 (6), 209 (5), 136 (6), 77 (51), 51 (22).

*3. General procedure for the preparation of alkynylaldehydes **4a-c***

Triethylamine (5.06 g, 6.95 mL, 50 mmol), the appropriate arylalkyne **3** (**3a**: ethynylbenzene, **3b**: 3-ethynylthiophene, **3c**: 1-hexyne) (15 mmol), $Pd(PPh_3)_2Cl_2$ (701 mg, 1 mmol) and CuI (380 mg, 2 mmol) were added under argon atmosphere to a solution of pyrazole **2** (2.746 g, 10 mmol) in dry dimethylformamide (30 mL) and the reaction mixture was stirred at 80°C for 2 h. Then the solvents were evaporated under reduced pressure, the residue was dissolved in CH_2Cl_2 (20 mL), water (40 mL) was added, and the mixture was extracted with CH_2Cl_2 (3×30 mL). The combined organic layers were washed with brine and dried over anhydrous Na_2SO_4. The solvent was evaporated and the residue was purified by column chromatography (SiO_2, EtOAc/light petroleum 1:7) to afford compounds **4** as the less retarded fractions and occasionally small amounts of compounds **8** as the more retarded ones. For the synthesis of **4c** the reaction mixture was stirred at 50°C for 1.5 h and for the chromatographic separation the eluent acetone/*n*-hexane 1:10 was used.

*3.1. 1-Phenyl-5-(phenylethynyl)-3-(trifluoromethyl)-1*H*-pyrazole-4-carbaldehyde (**4a**)*

Yield: 2.824 g (83%); light brown crystals: mp 77–78 °C. ^{1}H NMR (500 MHz, $CDCl_3$): δ 7.39 (m, 2H, CPh H-3,5), 7.45 (m, 1H, CPh H-4), 7.51 (m, 2H, CPh H-2,6), 7.52 (m, 1H, NPh H-4), 7.57 (m, 2H, NPh H-3,5), 7.82 (m, 2H, NPh H-2,6), 10.2 (q, *J* = 0.7 Hz, 1H, C*H*O) ppm. ^{13}C NMR (125 MHz, $CDCl_3$): δ 75.4 (*C*≡CPh), 102.3 (^{3}J = 5.5 Hz, C≡*C*Ph), 120.2 (q, $^{1}J_{CF3}$ = 270.6 Hz, *C*F_3), 120.4 (CPh C-1), 122.3 ($^{2}J_{C\text{-}4,CHO}$ = 27.6 Hz, $^{3}J_{C\text{-}4,CF3}$ = 0.8 Hz, C-4), 124.1 (NPh C-2,6), 128.7 (CPh C-3,5), 129.3 (NPh C-3,5), 129.6 (NPh C-4), 130.4 (CPh C-4), 130.6 ($^{3}J_{C\text{-}5,CHO}$ = 2.8 Hz, C-5), 132.0 (CPh C-2,6), 138.0 (NPh C-1), 142.0 (q, $^{2}J_{C\text{-}3,CF3}$ = 39.9 Hz, $^{3}J_{C\text{-}3,CHO}$ = 2.9 Hz, C-3), 181.9 ($^{1}J_{CHO}$ = 181.8 Hz, *C*HO) ppm. ^{15}N NMR (50 MHz, $CDCl_3$): δ −159.0 (N-1) ppm, N-2 not detected. ^{19}F NMR (470 MHz, $CDCl_3$): δ −61.6 (C*F_3*) ppm. IR (KBr): ν = 2220 (C≡C), 1682 (C=O) cm^{-1}. EIMS, *m/z* (rel. int.): 340 (33) $[M]^+$, 339 (100) $[M\text{-}H]^+$, 263 (34), 253 (20), 77 (60), 51 (31). HRMS (ESI), *m/z*: calcd. for $C_{19}H_{11}F_3N_2OH^+$ 341.0896 $[M+H]^+$; found 341.0895.

*3.2. 1-Phenyl-5-(3-thienylethynyl)-3-(triflouromethyl)-1*H*-pyrazole-4-carbaldehyde (**4b**)*

Yield: 2.875 g (83%); light brown crystals; mp 100–102 °C. ^{1}H NMR (500 MHz, $CDCl_3$): δ 7.17 (dd, $^{3}J_{Th\ H\text{-}4,Th\ H\text{-}5}$ = 5.0 Hz, $^{4}J_{Th\ H\text{-}2,Th\ H\text{-}4}$ = 1.2 Hz, 1H, Th H-4), 7.35 (dd, $^{3}J_{Th\ H\text{-}4,Th\ H\text{-}5}$ = 5.0 Hz, $^{4}J_{Th\ H\text{-}2,Th\ H\text{-}5}$ = 3.0 Hz, 1H, Th H-5), 7.51 (m, 1H, Ph H-4), 7.56 (m, 2H, Ph H-3,5), 7.65 (dd, $^{4}J_{Th\ H\text{-}2,Th\ H\text{-}5}$ = 3.0 Hz, $^{4}J_{Th\ H\text{-}2,Th\ H\text{-}4}$ = 1.2 Hz, 1H, Th H-2), 7.80 (m, 2H, Ph H-2,6), 10.15 (q, *J* = 0.8 Hz, 1H, C*H*O) ppm. ^{13}C NMR (125 MHz, $CDCl_3$): δ 75.2 (s, *C*≡CTh), 97.7 (m, C≡*C*Th), 119.6 ($^{2}J_{Th\ C\text{-}3,Th\ H\text{-}2}$ = 3.2 Hz, $^{2}J_{Th\ C\text{-}3,Th\ H\text{-}4}$ = 5.0 Hz, $^{3}J_{Th\ C\text{-}3,Th\ H\text{-}5}$ = 11.2 Hz, Th C-3), 120.2 (q, $^{1}J_{CF3}$ = 270.5 Hz, *C*F_3), 122.3 ($^{2}J_{C\text{-}4,CHO}$ = 27.6 Hz, $^{3}J_{C\text{-}4,CF3}$ = 0.8 Hz, C-4), 124.1 (Ph C-2,6), 126.3 ($^{1}J_{Th\ C\text{-}5,Th\ H\text{-}5}$, $^{2}J_{Th\ C\text{-}5,Th\ H\text{-}4}$, $^{3}J_{Th\ C\text{-}5,Th\ H\text{-}2}$ = higher order, not detectable, Th C-5), 129.2 (Ph C-3,5), 129.46 ($^{1}J_{Th\ C\text{-}4,Th\ H\text{-}4}$ = 171.7 Hz, $^{2}J_{Th\ C\text{-}4,Th\ H\text{-}5}$ = 5.5 Hz, $^{3}J_{Th\ C\text{-}4,Th\ H\text{-}2}$ = 8.2 Hz, Th C-4), 129.54 (Ph C-4), 130.6 (C-5), 132.1 ($^{1}J_{Th\ C\text{-}2,Th\ H\text{-}2}$ = 188.9 Hz, $^{3}J_{Th\ C\text{-}2,Th\ H\text{-}4}$ = 8.4 Hz, $^{3}J_{Th\ C\text{-}2,Th\ H\text{-}5}$ = 4.8 Hz, Th C-2), 138.0 (Ph C-1), 142.0 (q, $^{2}J_{C\text{-}3,CF3}$ = 39.7 Hz, $^{3}J_{C\text{-}3,CHO}$ = 2.9 Hz, C-3), 181.9 ($^{1}J_{CHO}$ = 181.9 Hz, *C*HO) ppm. ^{15}N NMR (50 MHz, $CDCl_3$): δ −159.1 (N-1) ppm, N-2 not detected. ^{19}F NMR (470 MHz, $CDCl_3$): δ −61.5 (C*F_3*) ppm. EIMS, *m/z* (rel. int.): 346 (48) $[M]^+$, 345 (100) $[M\text{-}H]^+$, 77 (20), 51 (30). HRMS (ESI), *m/z*: calcd. for $C_{17}H_9F_3N_2OSH^+$ 347.0460 $[M+H]^+$; found 347.0462.

*3.3. 5-(1-Hexyn-1-yl)-1-phenyl-3-(trifluoromethyl)-1*H*-pyrazole-4-carbaldehyde (**4c**)*

Yield: 1.890 g (59%); brownish oil. ^{1}H NMR (400 MHz, $CDCl_3$): δ 0.91 (t, *J* = 7.4 Hz, 3H, C*H_3*CH_2), 1.39 (m, 2H, C*H_2*CH_3), 1.57 (m, 2H, CH_3CH_2C*H_2*), 2.49 (t, *J* = 7.0 Hz, 2H, C≡CC*H_2*), 7.50 (m, 1H, Ph H-4), 7.52 (m, 2H, Ph H-3,5), 7.73 (m, 2H, Ph H-2,6), 10.6 (q, *J* = 0.7 Hz, 1H, C*H*O) ppm. ^{13}C NMR (100 MHz, $CDCl_3$): δ 13.4 (*C*H_3CH_2), 19.4 (C≡C*C*H_2), 21.9 (*C*H_2CH_3), 29.7 (*C*$H_2CH_2CH_3$), 67.2 ($^{3}J_{C\equiv CCH2}$ = 4.4 Hz, *C*≡CCH_2), 105.5 (C≡*C*CH_2), 120.2 (q, $^{1}J_{CF3}$ = 270.4 Hz, *C*F_3), 122.2 ($^{2}J_{C\text{-}4,CHO}$ = 27.3 Hz, $^{3}J_{C\text{-}4,CF3}$ = 0.9 Hz, C-4), 124.2 (Ph C-2,6), 129.1 (Ph C-3,5), 129.4 (Ph C-4), 131.6 (C-5), 138.0 (Ph C-1), 141.6 (q, $^{2}J_{C\text{-}3,CF3}$ = 39.8 Hz, $^{3}J_{C\text{-}3,CHO}$ = 3.4 Hz, C-3), 182.1 ($^{1}J_{CHO}$ = 181.4 Hz, *C*HO) ppm. ^{15}N NMR (40 MHz,

$CDCl_3$): δ −158.8 (N-1) ppm, N-2 not detected. ^{19}F NMR (376 MHz, $CDCl_3$): δ −61.9 (C*F*$_3$) ppm. EIMS, *m/z* (rel. int.): 320 (0.4) $[M]^+$, 278 (20), 277 (53), 162 (36), 105 (54), 92 (22), 91 (100), 79 (42), 78 (45), 77 (58), 65 (20), 63 (21), 51 (20), 41 (37). HRMS (ESI), *m/z*: calcd. for $C_{19}H_{12}F_3N_3H^+$ 321.1209 $[M+H]^+$; found 321.1210.

*3.4. 5-(2-Oxo-2-phenylethyl)-1-phenyl-3-(trifluoromethyl)-1*H*-pyrazole-4-carbaldehyde (**8a**)*

Yield: 286 mg (8%), brownish oil. ^{1}H NMR (500 MHz, $CDCl_3$): δ 4.68 (s, 2H, C*H*$_2$CO), 7.46 (m, 2H, NPh H-2,6), 7.48 (m, 3H, NPh H-3,4,5), 7.49 (m, 2H, CPh H-3,5), 7.62 (m, 1H, CPh H-4), 7.96 (m, 2H, CPh H-2,6), 10.09 (q, *J* = 0.9 Hz, 1H, C*H*O) ppm. ^{13}C NMR (125 MHz, $CDCl_3$): δ 36.4 (*C*H$_2$CO), 118.5 ($^2J_{C\text{-}4,CHO}$ = 26.2 Hz, C-4), 120.9 (q, $^1J_{CF3}$ = 270.4 Hz, *C*F$_3$), 125.8 (NPh C-2,6), 128.3 (CPh C-2,6), 128.8 (CPh C-3,5), 129.7 (NPh C-3,5), 130.2 (NPh C-4), 133.9 (CPh C-4), 135.8 (CPh C-1), 137.0 (NPh C-1), 142.8 (C-5), 143.6 (q, $^2J_{C\text{-}3,CF3}$ = 38.9 Hz, C-3), 184.3 ($^1J_{CHO}$ = 181.6 Hz, $^4J_{CHO,CF3}$ = 1.7 Hz, *C*HO), 193.5 (*C*O) ppm. ^{15}N NMR (50 MHz, $CDCl_3$): δ −157.2 (N-1) ppm, N-2 not detected. ^{19}F NMR (470 MHz, $CDCl_3$): δ −59.5 (C*F*$_3$) ppm. HRMS (ESI), *m/z*: calcd. for $C_{19}H_{13}F_3N_3O_2H^+$ 359.1002 $[M+H]^+$; found 359.1000.

*3.5. 5-(2-Oxohexyl)-1-phenyl-3-(trifluoromethyl)-1*H*-pyrazole-4-carbaldehyde (**8c**)*

Yield: 338 mg (10%), yellowish oil. ^{1}H-NMR (400 MHz, $CDCl_3$): δ 0.90 (t, *J* = 7.1 Hz, 3H, C*H$_3$*CH$_2$), 1.28 (m, 2H, C*H$_2$*CH$_3$), 1.58 (m, 2H, CH$_3$CH$_2$C*H$_2$*), 2.60 (m, 2H, COC*H$_2$*CH$_2$), 4.06 (s, 2H, C*H$_2$*CO), 7.41 (m, 2H, Ph H-2,6), 7.52 (m, 3H, Ph H-3,4,5), 10.07 (q, *J* = 0.9 Hz, 1H, C*H*O) ppm. ^{13}C NMR (100 MHz, $CDCl_3$): δ 13.7 (*C*H$_3$CH$_2$,), 22.1 (*C*H$_2$CH$_3$), 25.5 (*C*H$_2$CH$_2$CH$_3$), 39.8 (*C*H$_2$CO), 42.9 (CO*C*H$_2$CH$_2$), 118.2 (C-4), 120.8 (q, $^1J_{CF3}$ = 270.4 Hz, CF$_3$) 125.9 (Ph C-2,6), 129.6 (Ph C-3,5), 130.2 (Ph C-4), 138.1 (Ph C-1), 142.6 (C-5), 143.5 (q, $^2J_{C3,CF3}$ = 38.9 Hz, C-3), 184.3 (*C*HO), 204.2 (*C*O). ^{15}N NMR (40 MHz, $CDCl_3$): δ −157.6 (N-1) ppm, N-2 not detected. ^{19}F NMR (376 MHz, $CDCl_3$): δ −59.6 (d, *J* = 0.9 Hz, C*F*$_3$) ppm. HRMS (ESI), *m/z*: calcd. for $C_{17}H_{17}F_3N_2O_2H^+$ 339.1315 $[M+H]^+$; found 339.1313.

*4. General procedure for the preparation of pyrazolo[4,3-*c*]pyridines **5a-c***

Method A: The appropriate 5-alkynylpyrazole **4a-c** (1 mmol) was dissolved in dimethylformamide (12 mL) and *tert*-butylamine (730 mg, 10 mmol) was added. The reaction was performed under microwave irradiation conditions with an Anton Paar Synthos 3000 reactor and P-Program (800 W, p-Rate 2.0 bar s^{-1}, IR: 150°C, p: 80 bar for 2 h). After completion, the solvents were removed under reduced pressure and the residue was purified by column chromatography (SiO_2, EtOAc/light petroleum 1:10).

Method B: Pyrazole **2** (275 mg, 1 mmol) was dissolved in dimethylformamide (12 ml). The appropriate arylacetylene **3a-c** (2 mmol), $Pd(PPh_3)_2Cl_2$ (42 mg, 0.06 mmol) and *tert*-butylamine (730 mg, 10 mmol) were added under argon. The reaction was performed under

microwave irradiation conditions with an Anton Paar Synthos 3000 reactor and P-Program (800 W, p-Rate 2.0 bar s^{-1}, IR: 150°C, p: 80 bar for 2 h). After completion, the solvents were removed under reduced pressure and the residue was purified by column chromatography (SiO_2, EtOAc/light petroleum 1:10).

Method C: A mixture of **8** (0.3 mmol) and ammonium acetate (150 mg, 1.93 mmol) in acetic acid (5 mL) was heated at 80 °C for 1 h with stirring. After the solution reached room temperature it was poured onto ice-water (20 mL). The mixture was exhaustively extracted with EtOAc, the combined organic phases were washed with H_2O and brine and dried over anhydrous Na_2SO_4. The solvents were removed under reduced pressure and the residue was subjected to column chromatography (SiO_2, EtOAc/light petroleum 1:10).

*4.1. 1,6-Diphenyl-3-(trifluoromethyl)-1*H*-pyrazolo[4,3-*c*]pyridine (**5a**)*

Yield: method A: 240 mg (71%), method B: 300 mg (89%), method C: 72 mg (71%); yellow crystals; mp 121–123 °C. ^{1}H NMR (500 MHz, $CDCl_3$): δ 7.45 (m, 1H, CPh H-4), 7.51 (m, 2H, CPh H-3,5), 7.52 (m, 1H, NPh H-4), 7.63 (m, 2H, NPh H-3,5), 7.74 (m, 2H, NPh H-2,6), 7.96 (d, J = 1.1 Hz, 1H, H-7), 8.04 (m, 2H, CPh H-2,6), 9.34 (s, 1H, H-4). ^{13}C NMR (125 MHz, $CDCl_3$): δ 101.4 ($^1J_{C\text{-}7,H\text{-}7}$ = 166.7 Hz, $^4J_{C\text{-}7,H\text{-}4}$ = 1.8 Hz, C-7), 117.4 (C-3a), 121.2 (q, $^1J_{CF3}$ = 269.6 Hz, *C*F_3), 123.4 (NPh C-2,6), 127.4 (CPh C-2,6), 128.6 (NPh C-4), 128.9 (CPh C-3,5), 129.3 (CPh C-4), 129.9 (NPh C-3,5), 136.7 (q, $^2J_{C\text{-}3,CF3}$ = 39.7 Hz, $^3J_{C\text{-}3,H\text{-}4}$ = 0.9 Hz, C-3), 138.2 (NPh C-1), 138.9 (CPh C-1), 144.1 ($^2J_{C\text{-}7a,H\text{-}7}$ = 1.1 Hz, $^3J_{C\text{-}7a,H\text{-}4}$ = 6.2 Hz, C-7a), 144.5 ($^1J_{C\text{-}4,H\text{-}4}$ = 185.8 Hz, C-4), 154.9 (C-6). ^{15}N NMR (50 MHz, $CDCl_3$): δ −184.5 (N-1), −84.1 (N-5) ppm, N-2 not detected. ^{19}F NMR (470 MHz, $CDCl_3$): δ −60.8 (d, J = 0.9 Hz, C*F_3*). EIMS, *m/z* (rel. int.): 340 (21), 339 (100) $[M]^+$, 338 (81) $[M\text{-}H]^+$, 270 (23). HRMS (ESI), *m/z*: calcd. for $C_{19}H_{12}F_3N_3H^+$ 340.1056 $[M+H]^+$; found 340.1059.

*4.2. 1-Phenyl-6-(3-thienyl)-3-(trifluoromethyl)-1*H*-pyrazolo[4,3-*c*]pyridine (**5b**)*

Yield: method A: 180 mg (52%), method B: 175 mg (51%); light brown crystals; mp 118–121 °C. ^{1}H NMR (400 MHz, $CDCl_3$): δ 7.42 (dd, $^3J_{Th\ H\text{-}4,Th\ H\text{-}5}$ = 5.1 Hz, $^4J_{Th\ H\text{-}2,Th\ H\text{-}5}$ = 3.1 Hz, 1H, Th H-5), 7.52 (m, 1H, Ph H-4), 7.63 (m, 2H, Ph H-3,5), 7.67 (dd, $^3J_{Th\ H\text{-}4,Th\ H\text{-}5}$ = 5.1, $^4J_{Th\ H\text{-}2,Th\ H\text{-}4}$ = 1.3 Hz, 1H, Th H-4), 7.73 (m, 2H, Ph H-2,6), 7.82 (d, J = 1.2 Hz, 1H, H-7), 8.04 (dd, $^4J_{Th\ H\text{-}2,Th\ H\text{-}5}$ = 3.1 Hz, $^4J_{Th\ H\text{-}2,Th\ H\text{-}4}$ = 1.3 Hz, 1H, Th H-2), 9.26 (qd, J = 1.2, 0.9 Hz, 1H, H-4). ^{13}C NMR (100 MHz, $CDCl_3$): δ 100.6 ($^1J_{C\text{-}7,H\text{-}7}$ = 166.6 Hz, $^4J_{C\text{-}7,H\text{-}4}$ = 1.8 Hz, C-7), 117.2 (C-3a), 121.2 (q, $^1J_{CF3}$ = 269.7 Hz, *C*F_3), 123.4 (Ph C-2,6), 124.7 ($^1J_{Th\ C\text{-}2,Th\ H\text{-}2}$ = 185.8 Hz, $^3J_{Th\ C\text{-}2,Th\ H\text{-}4}$ = 8.6 Hz, $^3J_{Th\ C\text{-}2,Th\ H\text{-}5}$ = 4.6 Hz, Th C-2), 126.1 ($^1J_{Th\ C\text{-}4,Th\ H\text{-}4}$ = 167.2 Hz, $^2J_{Th\ C\text{-}4,Th\ H\text{-}5}$ = 5.1 Hz, $^3J_{Th\ C\text{-}4,Th\ H\text{-}2}$ = 8.5 Hz, Th C-4), 126.7 ($^1J_{Th\ C\text{-}5,Th\ H\text{-}5}$ = 185.7 Hz, $^2J_{Th\ C\text{-}5,Th\ H\text{-}4}$ = 7.7 Hz, $^3J_{Th\ C\text{-}5,Th\ H\text{-}2}$ = 6.0 Hz, Th C-5), 128.6 (Ph C-4), 129.9 (Ph C-3,5), 136.8 (q, $^2J_{C\text{-}3,CF3}$ = 39.8 Hz, C-3), 138.3 (Ph C-1), 141.5 (Th C-3), 144.0 (C-7a), 144.6 ($^1J_{C\text{-}4,H\text{-}4}$ = 185.6 Hz, $^4J_{C\text{-}4,H\text{-}7}$ = 1.3 Hz, $^4J_{C\text{-}4,CF3}$ = 0.9 Hz, C-4), 150.7 (C-6). ^{15}N NMR (40 MHz, $CDCl_3$): δ −184.6 (N-1), −85.1 (N-5) ppm, N-2 not detected. ^{19}F NMR (376 MHz, $CDCl_3$): δ −60.8 (d, J = 0.9 Hz,

C*F*$_3$). EIMS, *m/z* (rel. int.): 346 (23), 345 (100) $[M]^+$, 344 (33) $[M-H]^+$, 339 (21), 77 (20). HRMS (ESI), *m/z*: calcd. for $C_{17}H_{10}F_3N_3SH^+$ 346.0620 $[M+H]^+$; found 346.0621.

*4.3. 6-Butyl-1-phenyl-3-(trifluoromethyl)-1*H*-pyrazolo[4,3-*c*]pyridine (**5c**)*

Yield: method B: 294 mg (92%), method C: 67 mg (70%); brownish oil. ^{1}H NMR (500 MHz, $CDCl_3$): δ 0.94 (t, *J* = 7.4 Hz, 3H, C*H*$_3$CH$_2$), 1.40 (m, 2H, C*H*$_2$CH$_3$), 1.76 (m, 2H, CH$_3$CH$_2$C*H*$_2$), 2.94 (m, 2H, CC*H*$_2$), 7.43 (s, 1H, H-7), 7.48 (m, 1H, Ph H-4), 7.59 (m, 2H, Ph H-3,5), 7.69 (m, 2H, Ph H-2,6), 9.19 (s, 1H, H-4). ^{13}C NMR (125 MHz, $CDCl_3$): δ 13.9 (*C*H$_3$CH$_2$), 22.5 (*C*H$_2$CH$_3$), 32.2 (*C*H$_2$CH$_2$CH$_3$), 38.4 (*C*CH$_2$), 103.1 ($^1J_{C-7,H-7}$ = 166.6 Hz, C-7), 116.8 (C-3a), 121.2 (q, $^1J_{CF3}$ = 269.6 Hz, *C*F$_3$), 123.2 (Ph C-2,6), 128.4 (Ph C-4), 129.8 (Ph C-3,5), 136.6 (q, $^2J_{C-3,CF3}$ = 39.7 Hz, C-3), 138.3 (Ph C-1), 143.8 ($^3J_{C-7a,H-4}$ = 6.3 Hz, C-7a), 144.2 ($^1J_{C-4,H-4}$ = 184.8 Hz, C-4), 159.7 (C-6). ^{15}N NMR (50 MHz, $CDCl_3$): δ −185.9 (N-1), −80.8 (N-5) ppm, N-2 not detected. ^{19}F NMR (470 MHz, $CDCl_3$): δ −60.8 (d, *J* = 0.8 Hz, C*F*$_3$). EIMS, *m/z* (rel. int.): 319 (1.4) $[M]^+$, 278 (16), 277 (100), 77 (23). HRMS (ESI), *m/z*: calcd. for $C_{17}H_{16}F_3N_3H^+$ 320.1369 $[M+H]^+$; found 320.1371.

*5. General procedure for the preparation of oximes **6a-c***

The appropriate pyrazole aldehyde **4a-c** (1 mmol) was dissolved in EtOH (5 mL) and hydroxylamine hydrochloride (70 mg, 1 mmol) and sodium acetate (136 mg, 1 mmol) were added. The reaction mixture was stirred at room temperature for 1 h, then it was poured into ice-cold water. The solid product was filtered off and purified by column chromatography (SiO_2, EtOAc/light petroleum 1:5).

*5.1. (*E*)-1-Phenyl-5-(phenylethynyl)-3-(trifluoromethyl)-1*H*-pyrazole-4-carbaldehyde oxime (**6a**)*

Yield 282 mg (79%); light yellow, crystals; mp 128–130 °C. ^{1}H NMR (500 MHz, $CDCl_3$): δ 7.35 (m, 2H, CPh H-3,5), 7.40 (m, 1H, CPh H-4), 7.46 (m, 2H, CPh H-2,6), 7.48 (m, 1H, NPh H-4), 7.54 (m, 2H, NPh H-3,5), 7.81 (m, 2H, NPh H-2,6), 8.29 (s, 1H, NC*H*), 8.92 (br s, 1H, O*H*). ^{13}C NMR (125 MHz, $CDCl_3$): δ 76.4 (s, *C*≡CPh), 101.3 (m, C≡*C*Ph), 116.3 ($^2J_{C-4,NCH}$ = 7.3 Hz, $^3J_{C-4,CF3}$ = 1.1 Hz, C-4), 120.7 (q, $^1J_{CF3}$ = 269.8 Hz, *C*F$_3$), 121.0 (CPh C-1), 124.1 (NPh C-2,6), 126.1 ($^3J_{C-5,NCH}$ = 4.7 Hz, C-5), 128.5 (CPh C-3,5), 129.07 (NPh C-4), 129.1 (NPh C-3,5), 129.8 (CPh C-4), 131.7 (CPh C-2,6), 138.5 (NPh C-1), 140.1 (q, $^2J_{C-3,CF3}$ = 38.5 Hz, $^3J_{C-3,NCH}$ = 3.5 Hz, C-3), 140.4 (N*C*H) ^{15}N NMR (50 MHz, $CDCl_3$): δ −160.0 (N-1), -15.6 (*N*OH) ppm, N-2 not detected. ^{19}F NMR (470 MHz, $CDCl_3$): δ −61.7 (C*F*$_3$). EIMS, *m/z* (rel. int.): 355 (12) $[M]^+$, 354 (23) $[M-H]^+$, 339 (27), 338 (50), 269 (27), 268 (23), 77 (100), 69 (59), 51 (44). HRMS (ESI), *m/z*: calcd. for $C_{19}H_{12}F_3N_3OH^+$ 356.1005 $[M+H]^+$; found 356.1003.

*5.2. (*E*)-1-Phenyl-5-(3-thienylethynyl)-3-(trifluoromethyl)-1*H*-pyrazole-4-carbaldehyde oxime **(6b)***

Yield 221 mg (61%); light brown crystals; mp 115–117 °C. ^{1}H NMR (500 MHz, CDCl$_3$): δ 7.13 (m, $^{3}J_{\mathrm{Th\ H\text{-}4,Th\ H\text{-}5}}$ = 5.0, $^{4}J_{\mathrm{Th\ H\text{-}2,Th\ H\text{-}4}}$ = 1.2 Hz, 1H, Th H-4), 7.31 (m, $^{3}J_{\mathrm{Th\ H\text{-}4,Th\ H\text{-}5}}$ = 5.0 Hz, $^{4}J_{\mathrm{Th\ H\text{-}2,Th\ H\text{-}5}}$ = 3.0 Hz, 1H, Th H-5), 7.48 (m, 1H, Ph H-4), 7.53 (m, 2H, Ph H-3,5), 7.59 (m, $^{4}J_{\mathrm{Th\ H\text{-}2,Th\ H\text{-}5}}$ = 3.0 Hz, $^{4}J_{\mathrm{Th\ H\text{-}2,Th\ H\text{-}4}}$ = 1.2 Hz, 1H, Th H-2), 7.78 (m, 2H, Ph H-2,6), 8.28 (s, 1H, NC*H*), 9.20 (br s, NO*H*). ^{13}C NMR (125 MHz, CDCl$_3$): δ 76.1 (s, *C*≡CTh), 96.8 (m, C≡*C*Th), 116.1 ($^{2}J_{\mathrm{C\text{-}4,NC}H}$ = 7.4 Hz, $^{3}J_{\mathrm{C\text{-}4,CF3}}$ = 1.1 Hz, C-4), 120.1 ($^{2}J_{\mathrm{Th\ C\text{-}3,Th\ H\text{-}2}}$ = 3.2 Hz, $^{2}J_{\mathrm{Th\ C\text{-}3,Th\ H\text{-}4}}$ = 4.9 Hz, $^{3}J_{\mathrm{Th\ C\text{-}3,Th\ H\text{-}5}}$ = 11.1 Hz, Th C-3), 120.7 (q, $^{1}J_{\mathrm{CF3}}$ = 269.8 Hz, *C*F$_3$), 124.1 (Ph C-2,6), 125.9 ($^{1}J_{\mathrm{Th\ C\text{-}5,Th\ H\text{-}5}}$ = 189.2 Hz, $^{2}J_{\mathrm{Th\ C\text{-}5,Th\ H\text{-}4}}$ = 5.9 Hz, $^{3}J_{\mathrm{Th\ C\text{-}5,Th\ H\text{-}2}}$ = 5.9 Hz, Th C-5), 126.0 ($^{3}J_{\mathrm{C\text{-}5,NC}H}$ = 4.8 Hz, C-5) 129.1 (Ph C-3,4,5), 129.5 ($^{1}J_{\mathrm{Th\ C\text{-}4,Th\ H\text{-}4}}$ = 171.1 Hz, $^{2}J_{\mathrm{Th\ C\text{-}4,Th\ H\text{-}5}}$ = 6.0 Hz, $^{3}J_{\mathrm{Th\ C\text{-}4,Th\ H\text{-}2}}$ = 8.3 Hz, Th C-4), 131.2 ($^{1}J_{\mathrm{Th\ C\text{-}2,Th\ H\text{-}2}}$ = 188.9 Hz, $^{3}J_{\mathrm{Th\ C\text{-}2,Th\ H\text{-}4}}$ = 8.4 Hz, $^{3}J_{\mathrm{Th\ C\text{-}2,Th\ H\text{-}5}}$ = 4.8 Hz, Th C-2), 138.5 (Ph C-1), 140.2 (q, $^{2}J_{\mathrm{C\text{-}3,CF3}}$ = 38.3 Hz, $^{3}J_{\mathrm{C\text{-}3,NC}H}$ = 3.3 Hz, C-3), 140.3 (N*C*H). ^{15}N NMR (50 MHz, CDCl$_3$): δ −159.9 (N-1), −16.9 (*N*OH) ppm, N-2 not detected. ^{19}F NMR (470 MHz, CDCl$_3$): δ −61.6 (C*F$_3$*). EIMS, *m/z* (rel. int.): 361 (17) [M]$^+$, 345 (30), 344 (100), 275 (47), 274 (32), 77 (49). HRMS (ESI), *m/z*: calcd. for $C_{17}H_{10}F_3N_3OSH^+$ 362.0569 [M+H]$^+$; found 362.0569.

*5.3. (*E*)-1-[5-(1-Hexyn-1-yl)-1-phenyl-3-(trifluoromethyl)-1*H*-pyrazole-4-carbaldehyde oxime **(6c)***

Yield 209 mg (64%); light brown crystals; mp 70–71 °C. ^{1}H NMR (400 MHz, CDCl$_3$): δ 0.89 (t, *J* = 7.3 Hz, 3H, C*H$_3$*CH$_2$), 1.37 (m, 2H, C*H$_2$*CH$_3$), 1.54 (m, 2H, CH$_3$CH$_2$C*H$_2$*), 2.46 (t, *J* = 7.1 Hz, 2H, C≡CC*H$_2$*), 7.46 (m, 1H, Ph H-4), 7.49 (m, 2H, Ph H-3,5), 7.72 (m, 2H, Ph H-2,6), 8.18 (s, 1H, NO*H*), 8.21 (q, *J* = 0.5 Hz, 1H, NC*H*). ^{13}C NMR (100 MHz, CDCl$_3$): δ 13.5 (*C*H$_3$CH$_2$), 19.4 (C≡C*C*H$_2$), 21.9 (*C*H$_2$CH$_3$), 29.8 (*C*H$_2$CH$_2$CH$_3$), 68.1 (*C*≡CCH$_2$), 104.0 (C≡*C*CH2), 115.9 (C-4), 120.8 (q, $^{1}J_{\mathrm{CF3}}$ = 296.9 Hz, *C*F$_3$), 124.1 (Ph C-2,6),126.9 (C-5), 128.9 (Ph C-4), 129.0 (Ph C-3,5), 138.6 (Ph C-1), 139.9 (q, $^{2}J_{\mathrm{C\text{-}3,CF3}}$ = 38.5 Hz, C-3), 140.7 ($^{1}J_{\mathrm{NCH}}$ = 168.8 Hz, N*C*H). ^{15}N NMR (40 MHz, CDCl$_3$): δ −160.2 (N-1), −15.3 (*N*OH) ppm, N-2 not detected. ^{19}F NMR (376 MHz, CDCl$_3$): δ −61.9 (d, *J* = 0.5 Hz, C*F$_3$*). EIMS, *m/z* (rel. int.): 335 (11) [M]$^+$, 293 (74), 277 (32), 276 (100), 77 (25). HRMS (ESI), *m/z*: calcd. for $C_{17}H_{16}F_3N_3OH^+$ 336.1318 [M+H]$^+$; found 336.1323.

*6. General procedure for the preparation of pyrazolo[4,3-*c*]pyridine 5-oxides **7a-c***

Method A: AgOTf (13 mg, 0.05 mmol) was added to a solution of the appropriate pyrazole oxime **6a-c** (1 mmol) in dichloromethane (5 mL) and the reaction mixture was stirred at room temp. for 1.5 h. The mixture was directly loaded onto a silica gel column and eluted with dichloromethane/methanol 9:1.

Method B: A mixture of oxime **9** (145 mg, 0.5 mmol), the appropriate alkyne **3** (0.6 mmol), Pd(OAc)$_2$ (5 mol %), PPh$_3$ (20 mol %), K_2CO_3 (276 mg, 3 mmol) in anhydrous DMF (6 mL) was treated under microwave irradiation (100 W) for 1 h at 80 °C. Then the solvent

was evaporated under reduced pressure, the residue was dissolved in dichloromethane (30 mL), water (20 mL) was added, and the mixture was extacted with dichloromethane (3×30 mL). The combined organic layers were washed with brine and dried oved anhydrous Na_2SO_4. The solvent was evaporated and the residue was purified by column chromatography (SiO_2, acetone/light petroleum 1:4).

*6.1. 1,6-Diphenyl-3-(trifluoromethyl)-1*H*-pyrazolo[4,3-*c*]pyridine 5-oxide (**7a**)*

Yield: method A: 336 mg (94%), method B: 53 mg (30%); colorless crystals; mp 126–128 °C. ^{1}H NMR (500 MHz, $CDCl_3$): δ 7.50 (m, 3H, CPh H-3,4,5), 7.51 (m, 1H, NPh H-4), 7.60 (m, 2H, NPh H-3,5), 7.69 (m, 2H, NPh H-2,6), 7.70 (d, J = 0.7 Hz, 1H, H-7), 7.75 (m, 2H, CPh H-2,6), 8.99 (t, J = 0.8 Hz, 1H, H-4). ^{13}C NMR (125 MHz, $CDCl_3$): δ 108.8 ($^1J_{C\text{-}7,H\text{-}7}$ = 170.9 Hz, $^4J_{C\text{-}7,H\text{-}4}$ = 1.2 Hz, C-7), 118.2 (C-3a), 120.7 (q, $^1J_{CF3}$ = 269.6 Hz, *C*F_3), 123.2 (NPh C-2,6), 128.4 (CPh C-3,5), 129.1 (NPh C-4), 129.6 (CPh C-2,6), 130.0 (CPh C-4), 130.1 (NPh C-3,5), 131.5 (C-4), 132.6 (CPh C-1), 134.8 (q, $^2J_{C\text{-}3,CF3}$ = 40.3 Hz, $^3J_{C\text{-}3,H\text{-}4}$ = 1.7 Hz, C-3), 135.6 ($^2J_{C\text{-}7a,H\text{-}7}$ = 1.8 Hz, $^3J_{C\text{-}7a,H\text{-}4}$ = 7.1 Hz, C-7a), 149.9 (C-6). ^{15}N NMR (50 MHz, $CDCl_3$): δ −182.2 (N-1), −99.7 (N-5) ppm, N-2 not detected. ^{19}F NMR (470 MHz, $CDCl_3$): δ −61.0 (d, J = 0.8 Hz, C*F*$_3$). EIMS, *m/z* (rel. int.): 355 (73), 354 (100), 327 (22), 326 (29), 301 (39), 77 (25). HRMS (ESI), *m/z*: calcd. for $C_{19}H_{12}F_3N_3OH^+$ 356.1005 $[M+H]^+$; found 356.1009

*6.2. 1-Phenyl-6-(3-thienyl)-3-(trifluoromethyl)-1*H*-pyrazolo[4,3-*c*]pyridine 5-oxide (**7b**)*

Yield: method A: 306 mg (85%), method B: 51 mg (28%); light brown crystals; mp 179–181 °C . ^{1}H NMR (500 MHz, $CDCl_3$): δ 7.42 (dd, $^3J_{Th\ H\text{-}4,Th\ H\text{-}5}$ = 5.1, $^4J_{Th\ H\text{-}2,Th\ H\text{-}5}$ = 3.1 Hz, 1H, Th H-5), 7.53 (m, 1H, Ph H-4), 7.62 (dd, $^3J_{Th\ H\text{-}4,Th\ H\text{-}5}$ = 5.1, $^4J_{Th\ H\text{-}2,Th\ H\text{-}4}$ = 1.3 Hz, 1H, Th H-4), 7.63 (m, 2H, Ph H-3,5), 7.69 (m, 2H, Ph H-2,6), 7.86 (d, J = 0.7 Hz, 1H, H-7), 8.48 (dd, $^4J_{Th\ H\text{-}2,Th\ H\text{-}5}$ = 3.1, $^4J_{Th\ H\text{-}2,Th\ H\text{-}4}$ = 1.3 Hz, 1H, Th H-2), 8.98 (quint, J = 0.7 Hz, 1H, H-4). ^{13}C NMR (125 MHz, $CDCl_3$): δ 106.8 ($^1J_{C\text{-}7,H\text{-}7}$ = 170.0 Hz, $^4J_{C\text{-}7,H\text{-}4}$ = 1.2 Hz, C-7), 117.5 (C-3a), 120.7 (q, $^1J_{CF3}$ = 269.5 Hz, $^4J_{CF3,H\text{-}4}$ = 0.7 Hz, *C*F_3), 123.2 (Ph C-2,6), 125.5 ($^1J_{Th\ C\text{-}5,Th\ H\text{-}5}$ = 186.8 Hz, $^2J_{Th\ C\text{-}5,Th\ H\text{-}4}$ = 7.4 Hz, $^3J_{Th\ C\text{-}5,Th\ H\text{-}2}$ = 6.0 Hz, Th C-5), 127.8 ($^1J_{Th\ C\text{-}4,Th\ H\text{-}4}$ = 169.6 Hz, $^2J_{Th\ C\text{-}4,Th\ H\text{-}5}$ = 5.4 Hz, $^3J_{Th\ C\text{-}4,Th\ H\text{-}2}$ = 8.5 Hz, Th C-4), 129.1 (Ph C-4), 129.5 ($^1J_{Th\ C\text{-}2,Th\ H\text{-}2}$ = 190.2 Hz, $^3J_{Th\ C\text{-}2,Th\ H\text{-}4}$ = 8.4 Hz, $^3J_{Th\ C\text{-}2,Th\ H\text{-}5}$ = 4.5 Hz, Th C-2), 130.1 (Ph C-3,5), 131.8 ($^1J_{C\text{-}4,H\text{-}4}$ = 192.2 Hz, C-4), 132.0 (Th C-3), 134.7 (q, $^2J_{C\text{-}3,CF3}$ = 40.3 Hz, $^3J_{C\text{-}3,H\text{-}4}$ = 1.9 Hz, C-3), 135.6 ($^2J_{C\text{-}7a,H\text{-}7}$ = 2.1 Hz, $^3J_{C\text{-}7a,H\text{-}4}$ = 7.2 Hz, C-7a), 137.8 (Ph C-1), 144.9 (C-6). ^{15}N NMR (50 MHz, $CDCl_3$): δ −183.0 (N-1), −101.0 (N-5) ppm, N-2 not detected. ^{19}F NMR (470 MHz, $CDCl_3$): δ −61.0 (d, J = 0.85 Hz, C*F*$_3$). EIMS, *m/z* (rel. int.): 362 (20), 361 (100) $[M]^+$, 333 (23), 332 (85), 328 (31), 288 (97), 77 (38). HRMS (ESI), *m*/*z*: calcd. for $C_{17}H_{10}F_3N_3OSH^+$ 362.0569 $[M+H]^+$; found 362.0574.

*6.3. 6-Butyl-1-phenyl-3-(trifluoromethyl)-1*H*-pyrazolo[4,3-*c*]pyridine 5-oxide (**7c**)*

Yield: method A: 284 mg (85%); brownish oil. ^{1}H NMR (400 MHz, $CDCl_3$): δ 0.98 (t, J = 7.3 Hz, 3H, CH_3CH_2), 1.48 (m, 2H, CH_2CH_3), 1.74 (m, 2H, $CH_3CH_2CH_2$), 3.04 (m, 2H, CCH_2), 7.52 (m, 2H, H-7, Ph H-4), 7.62 (m, 2H, Ph H-3,5), 7.67 (m, 2H, Ph H-2,6), 8.92 (s, 1H, H-4). ^{13}C NMR (100 MHz, $CDCl_3$): δ 13.8 (CH_3CH_2), 22.5 (CH_2CH_3), 28.8 ($CH_2CH_2CH_3$), 31.2 (CCH_2), 106.2 ($^1J_{C\text{-}7,H\text{-}7}$ = 169.3 Hz, C-7), 117.4 (C-3a), 120.7 (q, $^1J_{CF3}$ = 269.6 Hz, CF_3), 123.2 (Ph C-2,6), 129.0 (Ph C-4), 131.0 ($^1J_{C\text{-}4,H\text{-}4}$ = 191.3 Hz, C-4), 130.1 (Ph C-3,5), 134.8 (q, $^2J_{C\text{-}3,CF3}$ = 40.2 Hz, C-3), 135.6 ($^2J_{C\text{-}7a,H\text{-}7}$ = 1.7 Hz, $^3J_{C\text{-}7a,H\text{-}4}$ = 7.2 Hz, C-7a), 137.8 (Ph C-1), 153.2 (C-6). ^{15}N NMR (40 MHz, $CDCl_3$): δ −183.5 (N-1), −99.1 (N-5) ppm, N-2 not detected. ^{19}F NMR (376 MHz, $CDCl_3$): δ −61.1 (d, J = 0.9 Hz, CF_3). EIMS, *m/z* (rel. int.): 335 (12) $[M]^+$, 318 (23), 293 (100), 277 (71), 276 (95), 77 (31), 43 (20). HRMS (ESI), *m/z*: calcd. for $C_{17}H_{16}F_3N_3OH^+$ 336.1318 $[M+H]^+$; found 336.1321.

*6.4. (*E*)-1-[5-Chloro-1-phenyl-3-(trifluoromethyl)-1*H*-pyrazol-4-yl]-N-hydroxymethanimine (**9**)*

Carbaldehyde **2** (275 mg, 1 mmol) was dissolved in EtOH (5 mL) and hydroxylamine hydrochloride (70 mg, 1 mmol) and sodium acetate (136 mg, 1 mmol) were added. The reaction mixture was stirred at room temperature for 1 h, then it was poured into ice-cold water. The solid product was filtered off and purified by column chromatography (SiO_2, EtOAc/light petroleum 1:5) to afford 223 mg (77%) of colorless crystals of mp 161-162 °C. ^{1}H NMR (400 MHz, $CDCl_3$): δ 7.50−7.58 (m, 5H, Ph H), 8.19 (s, 1H, NC*H*), 8.74 (m, 1H, O*H*) ppm. ^{13}C NMR (100 MHz, $CDCl_3$): δ 110.7 ($^2J_{C\text{-}4,NCH}$ = 7.3 Hz, $^3J_{C\text{-}4,CF3}$ = 0.9 Hz, C-4), 120.5 (q, $^1J_{CF3}$ = 270.2 Hz, CF_3), 125.4(Ph C-2,6), 128.6 (C-5), 129.4 (Ph C-3,5), 129.8 (Ph C-4), 136.8 (Ph C-1), 139.7 ($^1J_{NCH}$ = 169.9 Hz, $^2J_{NCH,OH}$ = 10.2 Hz, N*C*H), 140.7 ($^2J_{C\text{-}3,CF3}$ = 38.8 Hz, $^3J_{C3,NCH}$ = 3.2 Hz, C-3) ppm. ^{15}N NMR (40 MHz, $CDCl_3$): δ −164.3 (N-1), −15.8 (*N*OH) ppm, N-2 not detected. ^{19}F NMR (376 MHz, $CDCl_3$): δ −62.3 (CF_3) ppm.
